# Health providers’ readiness for electronic health records adoption: A cross-sectional study of two hospitals in northern Ghana

**DOI:** 10.1371/journal.pone.0231569

**Published:** 2020-06-04

**Authors:** Abdul-Fatawu Abdulai, Fuseini Adam

**Affiliations:** 1 School of Nursing, University of British Columbia, Vancouver, British Columbia, Canada; 2 Department of Occupational and Environmental Health, Kwame Nkrumah University of Science and Technology, Kumasi, Ghana; 3 Nursing administration, Tamale Government Hospital, Ghana Health Service, Accra, Ghana; University of Ghana College of Health Sciences, GHANA

## Abstract

**Introduction:**

Electronic Health Records are receiving considerable attention as a valuable tool for managing clinical information. Despite the prospects of Electronic Health Records in developing countries, many pre-implementation assessments target organizational, managerial, and infrastructural readiness, but barely include a detailed examination of health provider readiness. Meanwhile, health provider readiness is a critical success factor for electronic health records in settings where the majority of the workforce is less likely to have basic computer skills. We sought to assess the readiness of health providers for electronic health records in Ghana.

**Materials and method:**

An institutional-based cross-sectional study was conducted among 350 health providers in northern Ghana from June-September 2019. Data were collected using a modified questionnaire on provider readiness. The mean overall readiness was calculated for each respondent. Providers with readiness score below the overall mean score were categorized as not being ready while those at or above the mean score were considered ready. Multiple linear regression was conducted to determine the factors that determine provider readiness.

**Results:**

Two hundred and nine health providers responded to the questionnaire (59.7 response rate). The mean overall readiness was 3.61 (SD = .76), mean core readiness was 3.74 (SD = .80), and mean engagement readiness was 3.47 (SD = .67). Using the average overall readiness score as the cut-off for determining being ready and not ready for electronic health records, overall readiness was 54.9%, core readiness was 67.2%, while engagement readiness was 43.1%. Age, sex, old employees compared to new employees, computer literacy, and knowledge of electronic health records were significant determinants of health providers’ readiness to adopt electronic health records.

**Conclusion:**

We observed that health providers were marginally ready for electronic health records adoption. While participants might have expressed dissatisfaction with paper-based records and expressed a desire for electronic health records, they expressed fear of the potential impact of computerized records. We proposed a robust informatics curriculum and capacity building workshops for improving provider readiness for electronic health records.

## Introduction

Electronic Health Records (EHR) are currently receiving considerable attention for sharing patient information, process improvement and optimization of patient outcomes in developing countries [1]. Due to its efficiency, many private and publicly funded health institutions are investing huge resources into the development of electronic health records [1]. Despite the potential of EHR in improving the quality, safety, and efficiency of patient care, a significant proportion of them (more than 50%) either fail or fail to properly support patient care [2–4]. This is worse in developing countries where health professionals, especially at the lower level and rural areas, are more likely to have high computer anxiety [5].

Readiness assessment has been recognized as a significant factor in the adoption and utilization of electronic health records [6]. Readiness assessment portrays a proper image of existing conditions and the preparedness of health institutions and health professionals to the new system [6]. However, many pre-implementation assessments have largely targeted organizational readiness, technical readiness, leadership, infrastructure, and financial readiness [2]. Few studies have reported on the readiness of health professionals before EHR implementation. Yet the success or failure of EHR in developing countries, to a large extent, depends on the readiness of health professionals to move from paper-based records to electronic records.

Readiness assessment can be defined as the preparedness of health institutions as well as health professionals to embrace changes brought by the introduction of computerized systems [7]. Health providers’ readiness assessment has been considered a critical success factor for the implementation of electronic systems [8, 9]. Electronic Health Records implementation is usually accompanied by perceptions of an increased workload associated with data entry, disruptions to workflow, fears in transitioning from paper records to computerized records, and changes in organizational culture required to effectively utilize EHR [10]. Conducting readiness assessment can reduce these fears and reduce the risk of losing substantial amounts of money, prevent delays and disappointments among staff and service users, reduce the risk of medical errors and motivate staff to support implementation strategies [11, 12].

### eHealth in Ghana

At present, patient records management in Ghana’s public hospitals remains exclusively paper-based, with most activities being carried out manually. Before 2010, the only form of electronic health management platform in Ghana was the District Health Information Management System (DHIMS). But unfortunately, DHIMS was mainly used in tracking key performance indicators by middle-level health managers, and it’s not available within hospital units for clinical care [13]. To ensure correspondent health records management for health providers at the clinical level, Ghana adopted the National eHealth Strategy in 2010, with an objective to implement a fully-functional electronic health record system in all major government hospitals across the country [14]. Since the adoption of this policy, several mini-projects have been piloted across hospitals to determine the feasibility of a large-scale EHR implementation [15].

Despite the Ghana government’s plans for large scale implementation, a literature search shows that only one study assessed the organizational and managerial readiness for EHR implementation in Ghana [16]. To the best of our knowledge, not a single readiness assessment targeting health providers have been conducted in Ghana and the whole West African sub-region. Meanwhile, early engagement of the end-users, training, and education remains virtually absent in EHR pre-implementation processes [17, 18]. The purpose of this study was to assess the preparedness of health professionals in embracing the impending implementation of electronic health records in Ghana using two major hospitals as case studies.

## Materials and method

Ethical clearance was obtained from the Institutional Review Board of the College of Basic and Applied Sciences of the University of Ghana (Approval number ECBAS 047/15-19). Verbal consent was sought, and participants were informed that completion of the questionnaire indicates consent to the study. During our interaction with the research participants in previous studies, we realized many of them were not comfortable signing a written consent form. This factor, in addition to the sheer volume of participants, made us opt for verbal consent. This was explained in the ethics protocol, and the written consent was waived by the ethics committee. Therefore, instead of providing written consent, we decided to read and explain the consent form to the providers during the clinical ward conferences before subsequently distributing the questionnaire.

An institutional-based cross-sectional study was conducted between June to September 2019 at Tamale teaching hospital and Tamale central hospital–both in northern Ghana. Tamale teaching hospital is the third-largest hospital in Ghana and serves as the main referral center for close to 5 million residents in the northern part of the country. Tamale central hospital, on the other hand, is the oldest and the second largest referral center in northern Ghana. Both facilities have a total health provider population of approximately 1500. We decided to assess health professionals’ readiness in these two hospitals because they are part of the hospitals earmarked for the planned nationwide roll-out of the national eHealth initiative, yet no informatics training nor any eHealth project has been piloted in either of these facilities.

### Sample size and sampling criteria

We estimated to recruit 240 heath providers from the two facilities. But to enhance the precision and accuracy of the findings, the number was increased to 350. Using a multistage sampling, three units were randomly selected from each hospital. Questionnaires were subsequently distributed to nurses in the selected units through convenience sampling. Because other health providers such as doctors, pharmacists, laboratory personnel were far lesser than nurses in the randomly selected units, the recruitment of these categories of health professionals was extended to other units beyond the randomized units.

### Research questionnaire and data collection procedure

A structured questionnaire adapted from Biruk et al. [19] “*readiness assessment instrument*” was used to solicit health providers’ responses on their readiness for EHR adoption. This instrument has been validated and used in other resource-constrained settings [20, 21]. In a South African study [22], Cronbach alpha was 0.864 and 0.910 for core readiness and engagement readiness, respectively. This is above the threshold of 0.7, thus indicating good reliability of the instrument. Exploratory factor analysis also showed that all the items load onto their respective factors (loading values ranged from 0.54 to 0.94).

Since the tool was initially developed for assessing provider and patient readiness for eHealth adoption, the original instrument was modified to fit the purpose of this study–assessing provider readiness for one aspect of eHealth (electronic health records). Items that exclusively assessed patient readiness were removed. For the engagement readiness dimension, items including, better provision of patient information, 24/7 hour access to medical services were removed. On core readiness dimension, client waiting time, patient needs for eHealth were also removed. The word “eHealth” was replaced with electronic health records to reflect the study context and purpose. The modified instrument was pretested with 15 health providers outside the study setting. The research team, an informatics specialist in developing countries as well as the heads of the selected units in the two hospitals, also assessed the instrument for content and face validity. Minor corrections, including using simple words and explanations of technical terms, were made to the modified instrument based on the results of the pretest and suggestions coming mainly from the unit heads. For simplicity, EHR was defined as electronic versions of paper medical files that allow health providers to input and access patient records from a computer. Negatively worded items were reverse coded, and a Cronbach’s alpha was calculated to determine the reliability and internal consistency of the items in our modified instrument.

The first part of the questionnaire comprised demographic information, knowledge of EHR, technical and organizational variables, as well as the relevance of EHR to health providers’ work. The second part contained questions on core readiness and engagement readiness. Core readiness was measured by health professionals’ dissatisfaction with paper records system (5 items) and their desire for an EHR (3 items), while engagement readiness was assessed based on the potential benefits and willingness to use EHR (4 items) and concerns about the negative impact of computerized records (5 items). Both core and engagement readiness was measured on a five-point Likert scale with one denoting strongly disagree to five indicating strongly agree. A higher score indicates better preparedness for EHR implementation.

The research assistant took advantage of weekly ward conferences organized in various units to explain the research to the participant. With the help of two research assistants who were trained on data collection procedures, the questionnaire were subsequently distributed to health professionals depending on their work schedule–morning, afternoon, and evening shift.

### Data analysis

All questionnaires were manually checked for completeness before entry into Microsoft excel. Data cleansing was done to ensure consistency and accuracy before being exported to SPSS version 24 for analysis. Descriptive statistics were used to describe the characteristics of the sample in terms of demographics and the appropriate variables. Categorical variables were presented in frequencies whiles continuous variables were presented in means and standard deviations. Participants’ readiness was assessed by calculating the overall readiness score for each respondent. To determine the overall readiness levels, the mean scores for each readiness dimension were calculated, and participants who scored below or above the mean overall readiness score were considered as not being ready (No) or ready (Yes) for EHR adoption, respectively. The linear association between the independent variables (age, sex, education, length of employment, computer literacy, presence of a computer at the workplace, knowledge of EHR, and professional affiliation) and the dependent variable (readiness score) was analyzed using Pearson correlation. Categorical variables with more than two levels were dummy-coded, thus bringing the number of independent variables to 8. Multiple linear regression was conducted to ascertain the determinants of health providers’ readiness for EHR implementation. Preliminary analyses were conducted to ensure there is no violation of the assumptions of normality, linearity, and multicollinearity.

## Results

Two hundred and nine (209) health providers from the two facilities responded to the survey. This corresponds to a 59.7% response rate. The mean age of the respondents was 28.88 years (SD = 6.45), and a median range of 19–59 years. Nurses form a majority (63.8%) of the respondents, followed by pharmacists (15%), midwives (14%), doctors (4.8%), and lab technicians (2.4%) were the least represented group. The majority of the participants (71.7%) were between the ages of 19–31, and a majority of them (59.2%) have worked for less than 2 years in their respective facilities. Table 1 presents the demographic characteristics of the respondents.

**Table 1:** Demographic characteristics of respondents.

Variable	Category	Number	Percentage
Sex	Male	103	49.8
	Female	104	50.2
Age	19–30	147	71.7
	31–40	49	23.9
	41 & above	9	4.4
Education	Diploma & below	84	58.6
	Degree and above	119	41.4
Profession	Medical doctor	10	4.8
	Midwifery	29	14
	Nurse	132	63.8
	Pharmacy	31	15
	Laboratory	5	2.4
Length of employment	≤ 6 months	52	25.9
	7-12months	38	18.9
	13–18 months	9	4.5
	19-23months	20	4
	≥ 24 months	82	40.8
Computer literacy	No	37	17.7
	Yes	172	82.3
Workplace Computer access	No	54	25.8
	Yes	155	74.2
Previous knowledge of EHR	No	117	56.25
	Yes	91	43.75
EHR will improve quality	No	5	2.43
	Yes	201	97.57

EHR = Electronic Health Records

### Demographics

#### Readiness assessments

We calculated the average readiness scores for core readiness, engagement readiness, as well as the average overall readiness across all respondents. Respondents with scores below the average overall readiness score were considered not ready, while respondents with scores at or above the average levels were considered ready–producing dichotomous responses (yes or no). The mean overall readiness was 3.61 (SD = .76), mean core readiness was 3.74 (SD = .80), median range = 1.1–5, and the mean engagement readiness was 3.47 (SD = .67), median = 1.5–5. Using readiness scores below or above the average overall readiness as the criterion for assessing readiness levels, 54.9% of the participants were considered ready for EHR implementation. From Table 2, 62.7% were assessed as being ready for core readiness, and for the engagement readiness dimension, 43.1% were assessed as being ready. While many respondents obtained higher core readiness, less than half obtained engagement readiness scores at or above the average overall readiness score. Table 2 shows the proportion of respondents across the readiness dimensions.

**Table 2:** Overall, core and engagement readiness levels.

Readiness	Core readiness	Engagement Readiness	Overall Readiness
Yes	62.7%	43.1%	54.9%
No	36.4%	56.9%	45.1%

### Determinants of health professionals’ readiness for an electronic health record

Table 3 reports the person correlation between health professionals’ readiness for EHR and age, sex, education, length of employment, computer literacy, presence of a computer at the workplace, knowledge of EHR, and professional affiliation. The correlation coefficients in Table 3 shows that being a midwife, a medical doctor, employed from 7–12 months, 13–18 months, and having access to a computer at the workplace were significantly correlated with health professionals’ readiness for EHR. Using all the variables in the Pearson correlation as the independent variables and overall readiness as the dependent variable in a multiple regression analysis, we found that younger health providers, males, old employees compared to those who worked for less than six months, computer literacy and knowledge of EHR were the significant predictors of health provider readiness–accounting for 27.4% of the variance. Table 4 provides a summary of the multiple regression results. Level of education, professional group, and providers who have worked for more than six months were not significant determinants of provider readiness for EHR adoption.

**Table 3:** Correlation between the independent variables and health provider readiness.

	1	2	3	4	5	6	7	8	9	10	11	12	13	14
**1.**Readiness	1.0													
**2.** Age	.04*	1												
**3.** Sex	-.09	.08	1											
**4.** Nurse	-.17	-.05*	-.13	1										
**5.** midwife	-.01*	-.11	-.04*	-.36	1									
**6.** Pharm	.12	.24	.40	-.35	.17	1								
**7.** Dr	-.04*	-.06	.03*	-.14	-.06	-.06	1							
**8.** education	-.06	.04*	.12	.14	.08	.14	.18	1						
**9.** ≥ 6 months	-.14	-.23	-.09	.14	.009	-.10	-.02	-.02	1					
**10.** 7–12 months	-.03*	-.13	-.09	.01*	.08	-.15	-.07	.06	-.27	1				
**11.** 13–18 months	.004**	.03*	.07	-.14	-.08	.11	.12	.03*	-.12	-.10	1			
**12.** 19–24 months	-.08	.17	.12	-.08	-.09	.19	-.05	-.005	-.18	-.15	-.06	1		
**13.** Com Lit	-.09	-.08	-.10	-.01*	.18	-.33	.07	-.22	.05*	.04	-.09	-.02*	1	
**14.** C. Access	.03*	.03*	-.08	-.16	.18	-.07	.09	-.09	-.09	-.06	-.03*	.04	.08	1
**15.** EHR Know	.02*	-.03*	-.07	-.10	.01*	-.15	.05	-.08	-.09	.11	-.04	.008**	.24	.01*

Comp lit = computer literacy

*p < 0.05

**p < 0.01, n = 209, C. Access = Computer access at work, EHR = Electronic Health Records

**Table 4:** Determinants of health provider readiness for EHR.

Variable	Category	*B*	*s*.*e*	β	*t*	*p*	*95%*	CI
Age		-.26	.11	-1.98	-2.2	.03*	-.011	-.017
Sex	Female	-.19	.09	-.15	-2.10	.03*	-.37	-.01
Profession	Nurse	-.16	.11	-.13	-1.48	.14	-.39	.05
	Midwife	-.05	.14	-.03	-.40	.68	-.35	.23
	Pharmacist	.23	.16	.13	1.42	.15	-.092	.56
	Doctor	-.31	.29	-.07	-1.06	.29	-.88	.26
	Ref Lab tech							
Education	Diploma & below	-.07	.09	-.05	-.75	.44	-.25	.11
Employment Length	≥ 6 months	-.22	.11	-.15	-2.05	.04*	-.45	-.009
	7–12 months	-.17	.12	-.11	-1.43	.15	-.41	.06
	13–18 months	-.16	.21	-.05	-.75	.45	-.59	.26
	19–24 months	.011	.10	.008	.11	.91	-.19	.21
	Ref more than 24							
Comp Lit Literacy	Yes	.35	.15	.17	2.32	.02*	.66	.98
Workplace access	Yes	-.19	.12	-.12	-1.6	.11	-.44	.051
EHR knowledge	Yes	.28	.09	.23	3.18	.002**	.11	.46

Comp lit = computer literacy

*p < 0.05

**p < 0.01, n = 209, s.e = standard error

## Discussion

The study assessed the readiness of health professionals in two hospitals in Ghana that are earmarked for the planned nationwide implementation of EHR. Health professionals were targeted because a key factor to the adoption and successful implementation of EHR depends on the readiness of health professionals [22]. Our assessment indicated an overall readiness level of 54.9%. This result was consistent with readiness assessment levels in other resource-constrained settings [23]. A majority (62.7%) of the respondents were considered to be ready for core readiness, while less than half (43.1%) were considered ready in the engagement readiness dimension. This implies that while health providers might have expressed their dissatisfaction with paper records systems and realized the need for an EHR (core readiness) they were seen as less actively engaged with EHR and were worried about the potential negative impact of computerized systems (engagement readiness). Measuring both engagement and core readiness helps assess the pros and cons of EHR, assess the risk, and determine the applicability of EHR in institutional contexts. The low engagement readiness (fears and concerns about the negative impact of EHR, and willingness for adoption) among health providers in the study context could partly be attributed to a lack of informatics curriculum in health professions education programs. In Ghana, only nursing education integrated informatics training into the nursing curriculum in 2016 [24], but graduates of that program have not yet been integrated into the health system. To ensure successful adoption and sustenance of EHR, health professions’ education needs to include a robust informatics curriculum that will prepare health providers on applications of ICTs in healthcare before they enter the job market. In the meantime, management should re-direct capacity building workshops towards enhancing the competencies of health providers on ICT.

In the multiple regression results in Table 4, the age of respondents, sex, length of employment, computer literacy, and knowledge of EHR were significant predictors of provider readiness for EHR adoption. Younger people were more likely to adopt EHR than older health providers. This supports the growing evidence that young people are more likely to accept eHealth technologies [25, 26]. This finding also implies that the incoming crop of health professionals may be more receptive to future electronic health initiatives in Ghana. Interestingly, health providers who worked for less than six months–who were also likely to be younger–were less likely than people who have worked for more than two years to adopt EHR. In other words, longer serving employees were more likely than new entrants to adopt EHR. This could probably be explained by the fact that those people might not have understood the terrain of the hospital settings and the tedious workflow imposed by paper-based records because they are new employees. This is in sharp contrast to other studies that showed that health providers with extensive years of experience were less likely to accept EHR [27].

Health providers with computer expertise or those who are comfortable using computers were more likely to express their readiness for EHR implementation. Likewise, providers with previous knowledge of EHR were more likely to adopt EHR. Readiness to accept information systems in healthcare is influenced by the level of computer expertise of health providers [28]. This supports the assertion that computer expertise has a direct influence on respondents’ perceptions of computer-based systems [29]. Studies conducted in other developing countries suggest that poor computer expertise was highly correlated with the non-readiness of health professionals to adopt EHR [21, 30, 31]. Given the low computer expertise of health providers in developing countries, the findings re-enforced the need for pre-implementation preparations to begin with adequate training of health providers on basic computer literacy and interoperability functionalities of EHR. Being a male gender was another significant determinant of provider readiness–supporting the assertion that males have more positive attitudes towards computer use than females [32]. These findings are important to pre-implementation readiness because the majority of healthcare providers in Ghana are made up of female nurses.

## Conclusion

In this study, 54.9% of participants were assessed as having an overall readiness for EHR implementation according to our defining criteria. While core readiness was higher–suggesting that providers were dissatisfied with paper records, engagement readiness was less than 50%, indicating that providers feared the impact of EHR. This means that providers might be interested in EHR but feared its potential impact. We also observed that younger health providers, males, working for longer periods compared to six months of work, knowledge of electronic health records, and computer literacy were the significant determinants of healthcare providers’ readiness for EHR implementation. We proposed IT capacity building for healthcare providers at the organizational level and a robust informatics curriculum in health professions education. EHR implementation–without corresponding provider expertise–would lead to costly and expensive failures that are often associated with information technology (IT) in healthcare [33].

## Limitations

Our study has several limitations. Aside from asking healthcare providers to provide a subjective assessment of their computer skills, the questionnaire in the core and engagement readiness assessment tool did not contain a detailed and objective assessment of computer literacy–a factor that significantly determines the adoption of computerized records. Further studies are needed for an objective and detailed assessment of the computer expertise of clinical care providers. This will determine the kind of computer training programs to be designed for improving the IT skills of health providers. Recruiting healthcare providers from only Northern Ghana might have biased our sample. However, the selected hospitals in this study have similar staff demographics to other hospitals that met the criteria for EHR implementation. While our findings may not be generalizable to other countries, it nevertheless highlighted the importance of improving human resource capacities in ICT and incorporating a comprehensive informatics education in health professions curricula in developing countries.

## Supporting information

S1 Data (DOCX)

S1 File (SAV)
