# Supplementary material for: Health providers’ readiness for electronic health records adoption: A cross-sectional study of two hospitals in northern Ghana
Source: PLoS One. 2020 Jun 4;15(6):e0231569. doi: 10.1371/journal.pone.0231569 (PMC7271985; doi:10.1371/journal.pone.0231569)
Supplement: S1 Data — (DOCX) [file pone.0231569.s001.docx]

**Questionnaire**

This study seek to assess the readiness of health professionals for a replacement of the current way of recording patient information on paper with an electronic health records. This study will improve the chances of a successful implementation of future Electronic Health records by identifying the factors that should be targeted during systems implementation. You should understand that your participation is entirely voluntary and you are not required to disclose your identity on the questionnaire. Data from this study will be handled in a private and confidential manner. This questionnaire will take approximately 10 minutes to complete.

In this study **Electronic Health Records (EH-Rs)** are electronic versions of paper medical files, allowing health professionals to input and access patient records into computers

**Section A: Demographic characteristics**

**I can explain further if you need further clarification**

1. What is your age…………..
2. Sex
3. Male B. Female What is your profession?
4. Medical doctor B. Midwifery C. Nursing D. Pharmacy E. Laboratory
5. Others (Please specify)………………………..
6. What is the name of your health facility………………………………………………..

But you can modify this based on the number of facilities

1. Educational status
2. Diploma and below B. Degree and above:
3. Length of employment
4. ≤ 6 months B. 7-12months C. 13-18 months D.19-24months E. ≥ 24 months
5. Are you computer literate?
6. Yes, B. No
7. Do you have access to computer at the workplace
8. Yes B. No
9. Do you have access to a computer at home
10. Yes B. No

**Section B: Knowledge and attitude towards EHR**

1. Do you have Knowledge of Electronic Health Records (EHR)?

Yes No

1. Do you have a positive feeling towards EHR implementation

Yes No

1. Do you have previous knowhow on EHR

Yes No

H

1. There is a presence of computer & other technologies to support EHR implementation

Yes No

1. I have some computer related skills

Yes No

1. EHR has importance:

Yes No

1. EHR will improve service quality

Yes No

1. EHR will save money & time

Yes No

1. EHR will increase patient satisfaction

Yes No

1. EHR will ensure easy access to patient records

Yes No

**Section C. Electronic Health Records Readiness (Modified from Biruk et al. 2014)**

The first part of the table below, each statement seek to assess your level of dissatisfaction with paper records and the need for electronic Health records. The other part assesses the potential benefits of EHR and your concern about the negative impact of EHR. From a scale of 1-5, please rate your level of agreement with each statement. 1 - Strongly Disagree 2 – Disagree 3 – Neutral 4 – Agree 5 – Strongly Agree

|  | **Dissatisfaction with paper records** | **1** | **2** | **3** | **4** | **5** |
| --- | --- | --- | --- | --- | --- | --- |
|  | **Dissatisfaction with paper records** |  |  |  |  |  |
| **Core Readiness** | There is inefficient documentation in paper records | 1 | 2 | 3 | 4 | 5 |
|  | I am dissatisfied with the completeness and accuracy of paper records | 1 | 2 | 3 | 4 | 5 |
|  | There is difficulty in sharing patient information with other departments using paper records | 1 | 2 | 3 | 4 | 5 |
|  |  |  |  |  |  |  |
|  | **Need for Electronic Health Records** |  |  |  |  |  |
|  | I am comfortable with the use of technology | 1 | 2 | 3 | 4 | 5 |
|  | I trust in managing patient records with ICT | 1 | 2 | 3 | 4 | 5 |
|  | I am willing to use ICT for patient records | 1 | 2 | 3 | 4 | 5 |
|  | **Potential benefits and willingness to accept EHR** |  |  |  |  |  |
| **Engagement readiness** | Ensure Efficient documentation of patient records | 1 | 2 | 3 | 4 | 5 |
|  | Protection of patient privacy | 1 | 2 | 3 | 4 | 5 |
|  | Promote better provision of patient information | 1 | 2 | 3 | 4 | 5 |
|  | Ensure sharing of information | 1 | 2 | 3 | 4 | 5 |
|  |  |  |  |  |  |  |
|  | **Concern about negative impact of EHR** |  |  |  |  |  |
|  | High investment and low reimbursement | 1 | 2 | 3 | 4 | 5 |
|  | Individual limitation of IT knowledge | 1 | 2 | 3 | 4 | 5 |
|  | Time wasting | 1 | 2 | 3 | 4 | 5 |
|  | Changes in workflow | 1 | 2 | 3 | 4 | 5 |
|  | Elimination of traditional communication routines | 1 | 2 | 3 | 4 | 5 |
